# Immune Effects of Corticosteroids in Sepsis

**DOI:** 10.3389/fimmu.2018.01736

**Published:** 2018-07-30

**Authors:** Nicholas Heming, Sivanthiny Sivanandamoorthy, Paris Meng, Rania Bounab, Djillali Annane

**Affiliations:** ^1^General Intensive Care Unit, Raymond Poincaré Hospital, Garches, France; ^2^U1173 Laboratory Inflammation and Infection, University of Versailles SQY-Paris Saclay – INSERM, Montigny-Le-Bretonneux, France

**Keywords:** glucocorticoids, mineralocorticoids, NF-κB, animal models, clinical trials, septic shock, sepsis, organ function

## Abstract

Sepsis, a life-threatening organ dysfunction, results from a dysregulated host response to invading pathogens that may be characterized by overwhelming systemic inflammation or some sort of immune paralysis. Sepsis remains a major cause of morbidity and mortality. Treatment is nonspecific and relies on source control and organ support. Septic shock, the most severe form of sepsis is associated with the highest rate of mortality. Two large multicentre trials, undertaken 15 years apart, found that the combination of hydrocortisone and fludrocortisone significantly reduces mortality in septic shock. The corticosteroids family is composed of several molecules that are usually characterized according to their glucocorticoid and mineralocorticoid power, relative to hydrocortisone. While the immune effects of glucocorticoids whether mediated or not by the intracellular glucocorticoid receptor have been investigated for several decades, it is only very recently that potential immune effects of mineralocorticoids *via* non-renal mineralocorticoid receptors have gained popularity. We reviewed the respective role of glucocorticoids and mineralocorticoids in counteracting sepsis-associated dysregulated immune systems.

## Introduction

**Figure 1:**
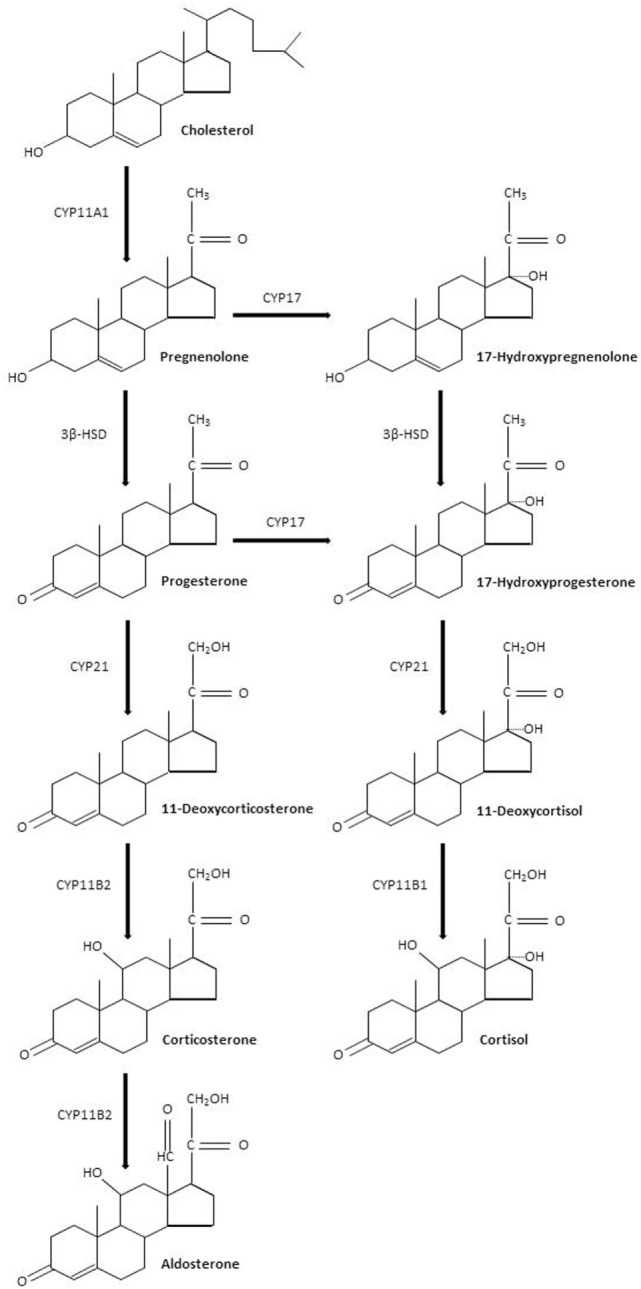
Corticosteroids biosynthesis. Abbreviations: CYP11A1, cholesterol desmolase; 3β-HSD, 3β hydroxysteroid dehydrogenase; CYP17, steroid 17α-hydroxylase; CYP21, steroid 21-hydroxylase; CYP11B2, aldosterone synthase; CYP11B1, steroid 11β-hydroxylase.

Sepsis is defined by a life-threatening organ dysfunction resulting from deregulated host response to invading pathogens (1). The host–pathogen interaction in sepsis is associated with an excessive response of the innate immune system leading to systemic inflammation and organ failure (2). This excessive inflammatory response coexists with compensatory anti-inflammatory signaling (3). An initial immune response occurs after recognition of pathogen- or damage-associated molecular patterns by specific cellular receptors, leading to cellular activation and systemic inflammation (4, 5). In practice, patients with sepsis may present with a hyperimmune response, typically around the time of admission when the infectious process is not fully under control, or with an immune suppression state, which tends to occur at a later time (3). The resolution of inflammation is also an active process, partly mediated by lipid mediators such as eicosanoids, which exhibit pro-resolving proprieties, and lead to tissue reparation (6). Then, knowing the time course of the immune response to sepsis is likely a key factor for the success of immunomodulatory interventions (7). Sepsis is a leading cause of mortality and morbidity, with annual prevalence of sepsis estimated at 31.5 million and the annual number of deaths at 5.3 million, worldwide (8, 9). The incidence of sepsis is steadily rising (10). Approximately half of sepsis survivors suffer from physical and psychological sequel, directly impacting their quality of life (11, 12). Treatment of sepsis is based on source control and organs support (13). Corticosteroids are produced by the adrenal glands lying at the superior pole of the kidneys. Corticosteroids are synthesized by adrenal cortical cells from esterified cholesterol and possess four carbon rings. Each step of corticosteroid biosynthesis is controlled by a specific enzyme (Figure 1). Corticosteroids are divided into mineralocorticoids, which preferentially affect salt and water balance while glucocorticoids preferentially affect sugar metabolism and sex hormones. The adrenal cortex is divided into the zona glomerulosa, the outermost layer beneath the capsule, which secretes mineralocorticoids, the zona fasciculata, which secretes glucocorticoids, and the innermost layer, the zona reticularis, which secretes sex hormones. We will hereafter describe the biological effects of glucocorticoids and mineralocorticoids, without further mentioning sex hormones. Corticosteroids are commonly categorized according to their glucocorticoid and mineralocorticoid power, relative to hydrocortisone (Table 1). Glucocorticoids exhibit immune-modulating proprieties, in part through interaction with NF-κB (14). Thus, glucocorticoids have been used to treat patients with severe infections for more than 50 years. Much less information is available regarding immune effects of mineralocorticoids, yet the combination of fludrocortisone to hydrocortisone significantly reduced mortality from septic shock (15, 16). We herein reviewed the immune effects of glucocorticoids and mineralocorticoids that may be relevant to the management of sepsis.

**Table 1:** Relative potencies of natural and synthetic steroids.

Compound	Glucocorticoid activity	Mineralocorticoid activity
**Natural steroids**		
Cortisol	1	1
Corticosterone	0.3	15
Aldosterone	0.3	3,000
Deoxycorticosterone	0.2	100
**Synthetic steroids**		
Cortisone	0.8	0.8
Fludrocortisone	10	125
Prednisone	4	0.8
Prednisolone	4	0.8
Methylprednisolone	5	0.5
Betamethasone	25	0
Dexamethasone	25	0

*Glucocorticoid and mineralocorticoid activity of natural and synthetic steroids, relative to cortisol*.

## Immune-Modulating Effects of Corticosteroids

### Glucocorticoids

#### Molecular Mechanisms of Action

**Figure 2:**
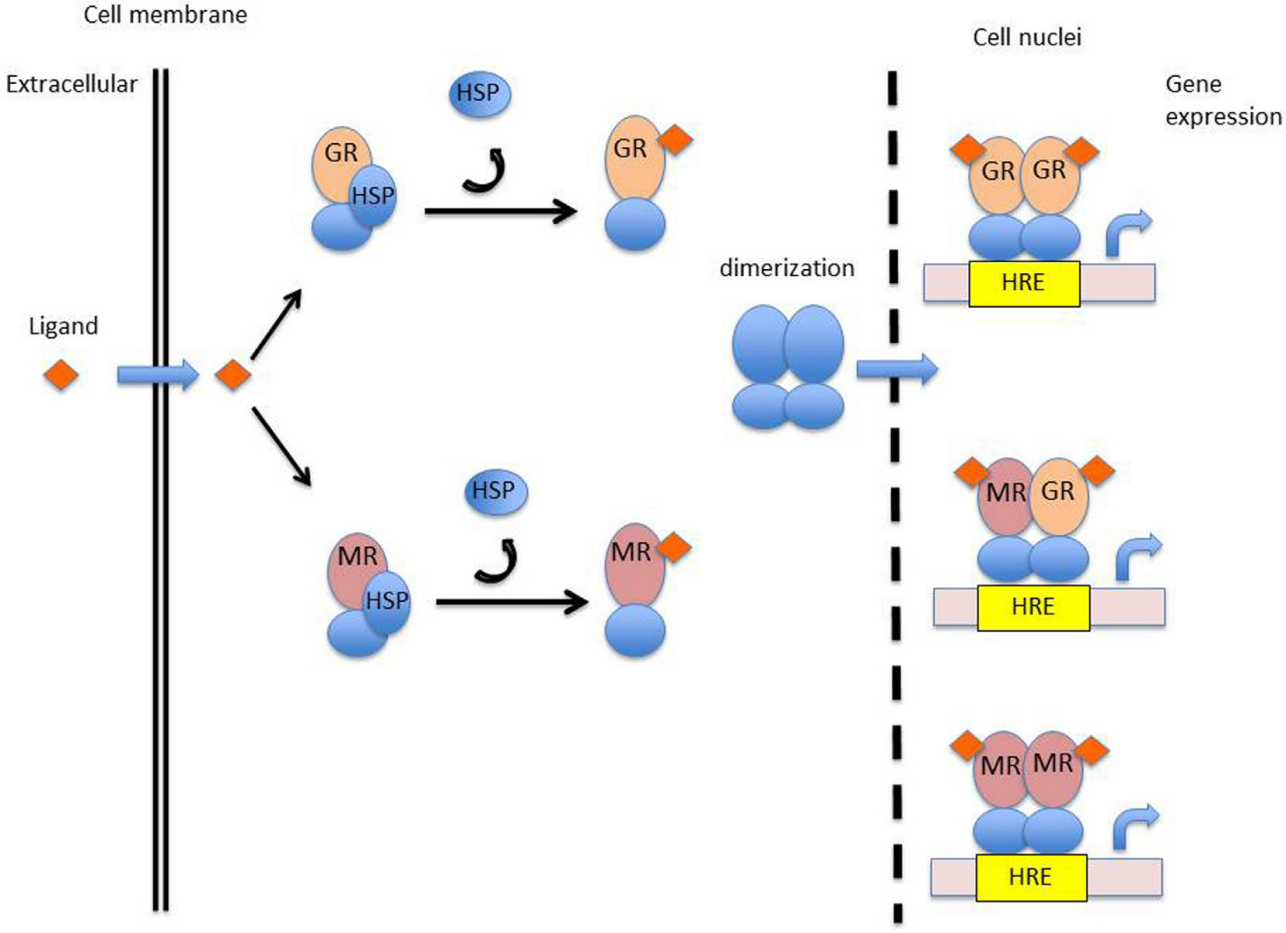
Mechanism of action of corticosteroids. Abbreviations: GR, glucocorticoid receptor; MR, mineralocorticoid receptor; HSP, heat-shock protein; HRE, hormonal response element.

Glucocorticoids have anti-inflammatory effects through the production of anti-inflammatory proteins and inhibition of pro-inflammatory proteins. Glucocorticoids bind to a specific intracellular receptor, the glucocorticoid receptor (GR). The GR is a transcription factor belonging to the nuclear receptor superfamily encoded on chromosome 5q31-31 (17). Glucocorticoid-regulated transcription factors are 94-kDa proteins, composed of several specific domains. A ligand-binding domain made up of 12 α-helices is involved in the recognition and binding of corticosteroids, a DNA-binding domain composed of two zinc fingers for interaction of the hormone–receptor complex with specific DNA sequences, and a trans-activating domain for binding of transcriptional factors (18). Unbound GR located in the cytoplasm of almost all cells, are stabilized by chaperone proteins such as heat-shock proteins 70, heat-shock protein 90 (Hsp90), and immunophilin (19). Upon binding with glucocorticoids, the GR dissociates from chaperone proteins and translocates into the nucleus. Within the nucleus, homodimers of the glucocorticoid–GR complex interact with specific DNA sequences (glucocorticoid responsive elements) of the regulatory region of target genes (Figure 2). The expression of genes modulated by the hormone–GR complex occurs through chromatin remodeling (20, 21). Chromatin consists of nucleosomes; DNA associated with core histone proteins. Quiescent genes are composed of tightly wound DNA around histone proteins, hampering the ability of RNA polymerases to bind to DNA and to produce mRNA. Core histones may be acetylated, modifying the structure of nucleosomes, loosening the chromatin, and ultimately enhancing gene expression. Transcription factors such as NF-κB activate histone acetyltransferases (HATs), leading to acetylation of core histones. By contrast, histone deacetylases (HDACs) induce a tightening of the chromatin, repressing target genes expression. Activated GR inhibit HATs and activate HDACs, overall repressing the expression of pro-inflammatory genes. For instance, the expression of the IRF3 transcription factor, implicated in interferon production and viral protection, is downregulated by glucocorticoids (22, 23). The GR–glucocorticoid complex also inhibits the production of pro-inflammatory proteins by sequestration of NF-κB within the cytosol (24). NF-κB is implicated in the production of pro-inflammatory cytokines (25, 26). In a resting state, inactive NF-κB is bound to IκBα. Upon cellular activation, NF-κB and IκBα dissociate and NF-κB translocates into the cellular nucleus. Glucocorticoids increase the expression of the inhibitory protein IκBα, thereby sequestering NF-κB (27). Glucocorticoids also induce the expression of glucocorticoid-induced leucine zipper (GILZ) which inhibits NF-κB (28) as well as the anti-inflammatory protein MAP kinase phosphatase 1, which inhibits nuclear translocation of transcription factor GATA-3 implicated in Th2 type cytokine expression (29). In addition, glucocorticoids promote the production of annexin 1, which inhibits the expression of phospholipase A2. Phospholipase A2 catabolizes the production of arachidonic acid-derived elements, including prostaglandins and leukotrienes, which are implicated in pain and inflammatory responses (26). Annexin 1 is also implicated in the resolution of inflammation as well as in the phagocytosis by macrophages of apoptotic neutrophils (25).

The genomic effects of glucocorticoids take place only after several hours, following nuclear translocation of activated GR and gene regulation (30). This latency is explained by the time needed for mRNA production, protein synthesis, and transport (31).

#### Effect of Glucocorticoids in Health

Glucocorticoids suppress the production of acute phase reactants and of chemokines implicated in leukocyte chemo-attraction (32, 33), thereby reducing leukocytes migration into inflamed areas. Glucocorticoids suppress the expression of endothelial-leukocyte adhesion molecule 1, intracellular adhesion molecule 1 (ICAM-1), and vascular adhesion molecule 1 (VCAM-1) opposing to leukocytes trafficking through the endothelium (34, 35). Glucocorticoids affect both the innate and adaptive arms of the immune system. Target immune cells include (1) myeloid cells; macrophages, monocytes, dendritic cells (tissue-resident DCs, migratory DCs, and plasmacytoid DCs), as well as granulocytes and (2) lymphocytes, including CD8, T helper 1 (Th1), Th2, and Th17 as well as Treg and B cells (14). Broadly speaking, glucocorticoids repress the maturation, differentiation and proliferation of leukocytes of all subtypes.

Glucocorticoids attenuate fever by reducing monocytes and macrophages production of interleukin (IL)-1, TNF, IL-8, and MCP-1 (36). Glucocorticoids reduce the number of monocytes/macrophages, dendritic cells, and eosinophil and basophil granulocytes (37). Despite reducing the number of monocytes/macrophages, the capacity of glucocorticoid-treated macrophages for phagocytosis seems unaltered or even improved (38). Neutrophil granulocytes are not affected by the increased apoptosis induced by glucocorticoids, possibly because of the specific production of an inactive isoform of the GR (GR-beta) (39). Indeed, after treatment by glucocorticoids, the number of circulating neutrophil granulocytes increases, through an increased release by the bone marrow associated with increased demargination. However, these leukocytes may be functionally less efficient. Glucocorticoid-treated circulating polymorphonuclear leukocytes exhibit decreased levels of L-selectin receptors (40). Dexamethasone induces the expression by polymorphonuclear leukocytes of a decoy receptor for IL-1 (41). Glucocorticoids stabilize the lysosomal membranes, greatly reducing the amount of proteolytic enzymes released by lysosomes. Dendritic cells treated by glucocorticoids produce increased levels of the anti-inflammatory cytokines IL-10 and TGF-β (42). Glucocorticoids reduce the membrane expression of MHC class II and Fc receptors (43, 44) and suppress antigen presenting to T cells (45).

Activation, proliferation, and production of immunoglobulins by B cell lymphocytes are depressed by glucocorticoids (46, 47). They deplete thymic stroma cells and T cells by apoptosis (48–50). Circulating T-cell numbers are reduced with a shift from a pro-inflammatory Th1 phenotype to an anti-inflammatory Th2 phenotype (51–54). Glucocorticoids suppress the production by lymphocytes of the pro-inflammatory cytokines IL-2, IL-4, IL-5, IL-13, and INF (55, 56).

### Mineralocorticoids

#### Molecular Mechanisms of Action

While the immune effects of glucocorticoids have been extensively investigated, those of mineralocorticoids have only recently gained attention. The biological activity of mineralocorticoids is mediated by interaction with a specific intracellular receptor, the mineralocorticoid receptor (MR). The MR is a transcription factors belonging to the nuclear receptor superfamily, encoded on chromosome 4, in the q31.1 region (57, 58). The structure of the mineralocorticoid-regulated transcription factor is highly similar to that of the GR and displays several specific domains, including a ligand-binding domain, a DNA-binding domain, and a trans-activating domain. The amino acid sequence of the DNA-binding domain of the GR and MR are approximately 94% similar, indicating that these two receptors may recognize and bind similar DNA sequences. The activated MR regulates the expression of a set of genes within target tissues, in a similar way to that of the activated GR. Unbound MR are stabilized in the cytoplasm by Hsp90. Activated MR will shed their chaperone proteins and translocate into the nucleus, form dimmers, and go on to recognize specific hormone recognizing elements of the DNA (Figure 2) (59). There is evidence that MR and GR may form functional heterodimers with specific properties (60, 61).

Surprisingly, cortisol and aldosterone bind the MR with equal affinity, indicating that the MR does not specifically recognize mineralocorticoids over glucocorticoids (57). However, the transcriptional response of the MR in response to aldosterone is approximately 100-fold higher than cortisol (62). Corticosteroid specificity is in part due to the activity of the type 2 isoenzyme of the 11β-hydroxysteroid dehydrogenase (11βHSD2), found in the kidney and in the colon and located near the MR. 11βHSD2 metabolizes glucocorticoids into an inactive derivative, cortisone. Since mineralocorticoids are unaffected by 11βHSD2 they are therefore able to interact with the MR (63). Therefore, the role of glucocorticoid binding of MR in non-epithelial tissues, which are devoid of 11βHSD2, is raised. The MR has several isoforms, some of which are able to bind both glucocorticoids and mineralocorticoids, while other isoforms bind exclusively mineralocorticoids (64). The density of GR and MR varies from one tissue to another. For instance, MR expression is higher than GR expression in the central nervous system; MR and GR are similarly expressed in the cardiovascular system while GR expression is higher than MR expression in the immune system (65). Finally, GR and MR also differ in their capacity to inhibit AP-1-mediated gene activation (66).

#### Effect of Mineralocorticoids on the Immune System in Health

Mineralocorticoid receptors play specific roles depending on their tissue expression. MRs located in the kidneys and the colon are implicated in NaCl reabsorption and K^+^ secretion (67, 68), where NaCl reabsorption is mediated by serum and glucocorticoid-induced kinase (SGK1), GILZ protein, and the epithelial sodium channel (69). Mineralocorticoid stimulation promotes the expression by endothelial cells of the VCAM-1, ICAM-1, and P-selectin membrane receptors, implicated in the adhesion of leukocytes to endothelial cells (70). In endothelial cells, mineralocorticoids also induce the production of reactive oxygen species *via* the activation of NADPH oxidase and Rac1 (71). In the brain, MRs are specifically located in the limbic system and are implicated in learning and memory (72). MRs are expressed in monocytes and macrophages (73), dendritic cells (74), and neutrophils (75). MR signaling in myeloid cells induces a pro-inflammatory response (76, 77). Indeed, macrophages exposed to mineralocorticoid agonists undergo a M1 type pro-inflammatory polarization associated with an increased production of TNF-α and of reactive oxygen species (78–80). In microglial cells, which are resident macrophages of the central nervous system, aldosterone activation induces an increased production of TNF-α and IL-6 in response to lipopolysaccharide stimulation (81). By contrast, MR knockout macrophages or macrophages treated by MR antagonists exhibit a M2 anti-inflammatory polarization (78). Mineralocorticoid agonists induce the activation of the mitogen-activated protein kinase pathway in dendritic cells, leading to the secretion of IL-6 and TGF-β1 (74). Mineralocorticoids indirectly lead to an increase in platelet cytosolic calcium concentrations, leading to platelet activation, thrombin formation, and platelet procoagulant activity (82, 83). Indeed, platelet cytosolic calcium entry is upregulated by the serum- and glucocorticoid-inducible kinase isoform SGK1, which is upregulated by mineralocorticoids (84). SGK1 upregulates IL-17-producing CD4+ helper T cells (Th17 cells). Th17 cells are dependent on IL-23 expression; SGK1 ensures the proper expression of the IL-23 receptor (85).

Mineralocorticoid receptor activation indirectly affects T lymphocyte phenotype. Indeed, dendritic cells activated by mineralocorticoid agonists impose a pro-inflammatory Th17 phenotype on CD4 T cells (74). Aldosterone stimulates IL-1β secretion by macrophages through NF-κB signaling and reactive oxygen species generation. Aldosterone also increases the expression of NLRP3, implicated in the formation of inflammasone and mature IL-1β in human peripheral blood mononuclear cells (86). Infusion of aldosterone in rodents results in elevated plasma IL-1β levels (86). Human blood mononuclear cells exposed to MR antagonists produce less cytokines, including TNF, IL-1α, IL-2, IL-6, INFγ, and GM-CSF (87). Aldosterone and MR agonists promote myocardial and kidney fibrosis (88, 89). Fludrocortisone, at high doses administered *in vivo*, paradoxically exhibits anti-inflammatory properties. Fludrocortisone inhibits histamine release by basophils (90) and IL-1 production by lung fragments (91).

## Immunomodulation in Sepsis

### Glucocorticoids

#### Animal Studies

The expression of the GR is upregulated in LPS-stimulated mouse macrophages and in mouse models of sepsis (92–94) (Table 2). However, others have shown that GR expression and protein levels decreased following a TNF challenge (95). The binding capacity of the GR decreases after an endotoxin challenge (94). The binding capacity of the GR may be altered through the action of nitric oxide (96, 97). LPS challenge in GR knockout mice induces higher mortality than in control animals (98). The endothelial GR regulates NF-κB in a model of endotoxin-induced sepsis (99). The deletion of the GR from endothelial cells, through the activation of NF-κB, is associated with higher mortality, higher nitric oxide levels, and higher levels of pro-inflammatory cytokines (TNF-α and IL-6) (99). In small animals with sepsis, high doses of dexamethasone and methylprednisolone significantly prolong survival (100–102). High doses of methylprednisolone continuously administered in a canine model of endotoxin shock also improve survival (103).

**Table 2:** Main effects of glucocorticoid or mineralocorticoid administration during sepsis.

Compound	Glucocorticoid	Mineralocorticoid
Small animal	– Improves survival (100–102)	– Restores the expression of the mineralocorticoid receptor (104) – Reduces the levels of plasma histamine, plasma serotonin, blood bradykinin, and plasma catecholamine concentration (105) – Improves the hemodynamic response (106) – Reduces mortality (107–109)

Large animal	– Improves survival (103)	– Improves survival and the hemodynamic response (110, 111) – Lowers IL-6 levels (111)

Man	– Reduces plasma levels of TNF-α (112) – Reduced levels of E-selectin sE-selectin (113, 114) – Decreases the number of eosinophils (115) – Decreases levels of phospholipase A (116) – Decreases levels of nitrite/nitrate, IL-6, IL-8, and markers of neutrophil activation (decreased expression of CD11b, CD64, and neutrophil elastase) (116) – Lowers whole blood production of IL-1 and IL-6 (113, 116–119) – Monocyte mHLA-DR levels are depressed (113, 118) – Decreases monocyte production of migration inhibitory factor (120) – Decreases the binding capacity of glucocorticoid receptor in neutrophils (121) – Improves the hemodynamic response and survival (122)	– Improves the hemodynamic response in the most severe subgroup of a pediatric population (123)

#### Studies in Humans

When LPS is administered to healthy subjects, the concomitant administration of hydrocortisone reduces plasma levels of TNF-α (112). The levels of E-selectin and of the soluble form of the receptor sE-selectin are also reduced following hydrocortisone therapy in sepsis (113, 114). During septic shock, hydrocortisone decreases the number of eosinophils (115), circulating levels of phospholipase A (116), serum levels of nitrite/nitrate, IL-6, IL-8, and markers of neutrophil activation (decreased expression of CD11b, CD64, and neutrophil elastase). Moreover, hydrocortisone lowers *ex vivo* whole blood production of IL-1 and IL-6 in response to LPS (113, 116–119). In hydrocortisone-treated patients with septic shock, monocyte mHLA-DR levels are depressed, while the capacity for phagocytosis of monocytes increases (113, 118). Glucocorticoids also attenuate LPS-stimulated monocyte production of migration inhibitory factor (120). In neutrophils of hydrocortisone-treated sepsis patients, the binding capacity of GR for glucocorticoid is reduced (121).

#### Clinical Trials

Short courses of high dose methylprednisolone or dexamethasone do not significantly reduce mortality and may even harm patients with sepsis (124–127). The introduction of the concept of sepsis-associated relative adrenal insufficiency in the 90s, led physicians and trialists to consider using prolonged courses of low doses of hydrocortisone (128). Several small size trials found that 200–400 mg of hydrocortisone per day for more than 3 days improved cardiovascular function in sepsis (117, 129, 130). GERINF05 was the first phase 3 trial that tested a prolonged course (7 days) of low to moderate doses (200 mg/day) of corticosteroids in septic shock with evidence of relative adrenal insufficiency (15). This trial found a significant improvement on survival and cardiovascular function in patients with septic shock and non-responders to a 250 µg ACTH test (Delta cortisol <9 μg/dl) (15). The CORTICUS trial could not reproduce the survival benefit of corticosteroids found in GERINF05 while confirming the benefit on cardiovascular homeostasis and organs function (131). A meta-analysis of the use of corticosteroids in sepsis published in 2015 concluded that corticosteroids reduced 28-day mortality, increased the rate of shock reversal, without increasing the risk of infection (122). More recently, the ADRENAL trial found no significant survival benefit from a continuous infusion of hydrocortisone in patients with septic shock (132). Nevertheless, the trial found that, when compared with placebo, hydrocortisone fasten the resolution of shock, shortened the duration of mechanical ventilation, and reduced the requirement for blood transfusion (132). In keeping with GERINF05 observations, the APROCCHSS trial found that the combination of hydrocortisone to fludrocortisone significantly reduced 90-day mortality (16). Likewise, corticosteroids hastened the resolution of shock and organs failure without causing major adverse events.

### Mineralocorticoids

#### Animal Studies

In models of chronic cardiovascular diseases, aldosterone is associated with increased vascular and cardiac oxidative stress, inflammation, and fibrosis (133). In models of acute cardiovascular diseases, both exogenous aldosterone and overexpression of the MR increased blood pressure (134, 135). Aldosterone plays a role in salt appetite (136), and in coping behavior (137). MRs, contrary to GRs, when activated, play a neural antiapoptotic role by differentially influencing genes of the bcl-2 family (138). Similar to the renal epithelium, aldosterone may also favor the clearance of alveolar fluid by type II epithelial cells (139). By extrapolation, these responses may be of benefit during sepsis. However, very little is known about the MR or its regulation during sepsis. In small animals, sepsis is associated with downregulation of the MR in endothelial cells (92, 104), which is restored by supplementation by exogenous MR (104). In endotoxin shock, aldosterone reduces plasma levels of histamine, serotonin, bradykinin, and catecholamine (105). MR agonists reduce mortality in endotoxin-challenged small animals (107–109). In large animal sepsis models, aldosterone levels correlate with the severity of shock and with mortality (140), and mineralocorticoid supplementation improved survival and hastened shock reversal when administered prior to a bacterial challenge (110). The early administration of glucocorticoids plus mineralocorticoids improves the outcome in large animals with sepsis. In the sickest animals, glucocorticoids plus mineralocorticoids treatment hastened shock reversal and lowered plasma IL-6 levels (111).

#### Clinical Studies

The plasma concentrations of aldosterone were found to be unexpectedly low in meningococcal sepsis compared with ICU admissions for other reasons (141). Lower than expected aldosterone levels have also been reported in adults’ septic shock (142, 143). Inappropriately low aldosterone levels during septic shock were associated with increased ICU length of stay, an increased incidence of acute kidney failure (144), and increased mortality (145). Low aldosterone levels occurred despite high renin levels, suggesting impaired adrenal synthesis of aldosterone. In addition, a subset of patients with sepsis did not increase aldosterone levels in response to ACTH stimulation (146). Mineralocorticoid levels were found to correlate with IL-6 levels in meningococcal sepsis (147). The expression of the MR is downregulated in human endothelial cells exposed to TNF-α (104). The MR agonist fludrocortisone is administered orally, and there is no currently available intravenous formulation. The pharmacokinetics of fludrocortisone were assessed in healthy subjects (148), and in septic shock patients (149). However, there are still too few data on the direct effects of mineralocorticoids in patients with sepsis. A retrospective study in a pediatric population found that hydrocortisone combined with fludrocortisone was associated with shorter duration of vasopressor support in the most severe patients (123). Finally, it remains unclear whether or not part of the survival benefit from corticosteroids observed in the GERINF05 (15) and APROCCHSS (16) trials are directly related to fludrocortisone.

Future research should start with an individual patient data meta-analysis of all trials comparing hydrocortisone and/or fludrocortisone against placebo. Then, next trial should be designed as a two-by-two factorial placebo-controlled trial comparing hydrocortisone versus fludrocortisone versus hydrocortisone plus fludrocortisone (150).

## Conclusion

In sepsis, there is sufficient evidence from animals and humans studies to support that glucocorticoids modulate innate immunity to promote the resolution of inflammation and organs failure. Much less is known about the immune effects of mineralocorticoids though increasing evidence from laboratory investigations suggested that they might favorably impact the outcome from sepsis. So far, survival benefits in adults with septic shock have been shown only for the combination of hydrocortisone plus fludrocortisone and not from the administration of hydrocortisone alone.

## Author Contributions

DA has conceived the manuscript. All the authors have contributed to the literature search and writing of the manuscript.

## Conflict of Interest Statement

The authors declare that the research was conducted in the absence of any commercial or financial relationships that could be construed as a potential conflict of interest.
